# Rare Genetic Diseases with Founder Effect in Roma Children

**DOI:** 10.3390/life16050738

**Published:** 2026-04-29

**Authors:** Simona Drobňaková, Mária Andrejková, Jana Šaligová, Ľudmila Potočňáková, Veronika Vargová, Milan Kuchta, Roman Beňačka, László Barkai

**Affiliations:** 1 Department of Paediatrics and Adolescent Medicine, Faculty of Medicine, Pavol Josef Šafárik University, 040 11 Kosice, Slovakia; veronika.vargova@upjs.sk (V.V.); milan.kuchta@upjs.sk (M.K.); 2 Children’s Faculty Hospital, Tr. SNP 1, 040 11 Kosice, Slovakia; maria.andrejkova@dfnkosice.sk (M.A.); jana.saligova@dfnkosice.sk (J.Š.); ludmila.potocnakova@dfnkosice.sk (Ľ.P.); 3 Department of Pathological Physiology, UPJS, Tr. SNP 1, 040 11 Kosice, Slovakia; roman.benacka@upjs.sk; 4 Physiological Controls Research Center, University Research and Innovation Center, Obuda University, Bécsi út 96, 1034 Budapest, Hungary

**Keywords:** rare diseases, founder effect, Roma ethnicity, children

## Abstract

(1) Background: The characteristics of rare diseases (RDs) vary considerably—not only between different disease types but also between individual patients with the same condition. In the Roma community, we analyzed the most frequent rare genetic disorders related to the founder effect. (2) Methods: This retrospective study, conducted between January 2019 and January 2025 at the Clinical Genetics and Metabolics Outpatient Clinic in Košice, included 61 patients aged from infancy to 25 years diagnosed with hypomyelinating leukodystrophy 14, pontocerebellar hypoplasia type 1B, neuronal ceroid lipofuscinosis 7, or TMEM70 deficiency. (3) Results: This study includes the largest known cohort of patients with hypomyelinating leukodystrophy 14 caused by the *UFM1* c.-273_-271delTCA mutation, predominantly affecting males (*n* = 17). The disorder is severe, with most patients dying before one year of age, and is characterized by inspiratory stridor, axial hypotonia, spastic quadriparesis, pseudobulbar signs, and microcephaly. In a separate group with pontocerebellar hypoplasia type 1B, six Roma patients (three males, three females) shared the same *EXOSC3* mutation. Diagnosis occurred at an average age of 8.8 months, and most children did not survive beyond three years. Common features included microcephaly, severe hypotonia, and spastic quadriplegia. Thirteen children from eight families were diagnosed with neuronal ceroid lipofuscinosis 7, all carrying the same *MFSD8* mutation. Symptoms typically began with psychomotor regression between ages 3 and 4, along with intellectual disability and seizures, which were more frequent in males. The mean age at diagnosis was 4.5 years, and eight children died before age nine. Finally, 25 patients with TMEM70 deficiency associated with Roma ancestry were identified, predominantly females, with a mean age of 9.95 years and the oldest patient aged 25. Four children died due to severe metabolic crises. Common findings included intellectual disability, global hypotonia, hypertrophic cardiomyopathy, epilepsy, and failure to thrive. (4) Conclusions: Most rare diseases are genetic and carry high morbidity and mortality, with no targeted therapies currently available. Their increased prevalence in the Roma population reflects founder effects and high consanguinity. Prenatal and newborn screening, along with voluntary carrier testing for couples, is essential for proactive health management.

## 1. Introduction

The characteristics of rare diseases (RDs) vary considerably—not only between different diseases but also between individual patients with the same condition. Within the European Union (EU), rare diseases are recognized as life-threatening, chronically disabling, and significant, including those of genetic origin [1]. According to Regulation (EC) No. 141/2000 of the European Parliament and of the Council of 16 December 1999 on orphan medicinal products, the definition of a rare disease is standardized in the European Union as a condition affecting fewer than 5 individuals per 10,000 people, which is equivalent to fewer than 1 in 2000 individuals. Additionally, the National Institute for Health and Care Excellence (NICE) classified ultra-rare diseases as those with a prevalence of less than 1 case per 50,000 people [2].

An estimated 27 to 36 million people in the EU are living with RDs, and there are currently between 6000 and 8000 different types [3]. According to the Global Genes Project, approximately 300 million people worldwide are affected by rare diseases, and the Institute for Rare Genomics reports that only around 400 rare diseases are currently considered curable [4]. In the EU, the average RD diagnostic process takes five years and can often be significantly longer.

Genetic factors are responsible for the majority of rare and ultra-rare diseases. A 2022 study published in the Orphanet Journal of Rare Diseases found that most ultra-rare disorders are caused by changes in a single gene. These genetic alterations can be inherited in either an autosomal dominant or autosomal recessive manner. Notably, as the rarity of the disease increases, it becomes more likely that it is caused by a recessive genetic change [5].

A special group of RDs is characterized by the founder effect, occurring mainly in the Roma population. Genetic drift is an intriguing and random process that influences allele and genotype frequencies, particularly in small or isolated populations. It typically occurs when a small subset of individuals separates from the larger population and reproduces in isolation, leading to potential random changes in allele frequencies. Notably, two significant examples of this important process arise when a small group establishes a new colony, and the bottleneck effect occurs when a population is substantially reduced before reproducing. While the exact impact of this effect may be challenging to predict due to its inherent randomness, it is possible to estimate the range of potential changes in allele frequencies. The effective population size—reflecting the number of reproductive individuals and the ratio of male to female gametes—plays an important role in this phenomenon [6].

The Roma population is the largest transnational ethnic minority in Europe. While they are traditionally known for their nomadic lifestyle, many have been settled for centuries. It was estimated that approximately 10 million Roma lived in Europe in 2019. However, some Roma groups and international human rights organizations claim that the actual number exceeds 14 million, which would represent about 1.5% to 1.8% of the total population of Europe. The majority of Roma communities are found in southeastern Europe, but significant populations are also spread across central and western Europe. Additionally, some Roma communities live outside Europe, mainly in the Middle East and the Americas [7,8]. The earliest genetic evidence supporting an Indian origin for the Roma people comes from studies that examine genetic markers in blood groups and specific founder mutations in the γ-sarcoglycan gene [9]. The discovery of several Mendelian mutations, which can be attributed to a “founder effect,” includes cases such as myasthenia gravis caused by the 1267delG mutation in the *CHRNE* gene [10]. This particular form of the inherited disorder has only been reported in individuals of Indian and Pakistani descent, further indicating an Indian origin for the Roma. Additionally, mutations in the *GJB2* gene (which are linked to non-syndromic hearing loss) and mutations in the *LTBP2* gene have been identified in the Roma population and are known to be highly prevalent in many Indian communities [11]. Research using genome-wide SNP arrays and sequencing has revealed that the Roma people have approximately 20–35% South Asian ancestry, primarily originating from northwest India [12]. The genetic ancestry of South Asians is complex and can be categorized into two components: Ancestral North Indian (ANI) and Ancestral South Indian (ASI) [13]. A general consensus regarding Roma ancestry has emerged from analyses of uniparental markers and genome-wide sequencing. These findings indicate that all examined Roma populations originate from a common founder population [14,15,16].

Carrier rates for certain Mendelian disorders in this ethnicity range from 5% to 15% [17]. In 2025, Quinn et al. identified a total of 90 distinct autosomal recessive disorders, which correspond to 91 unique phenotypes and 111 pathogenic disease variants in the Roma population that may be used for early diagnoses, which could significantly improve overall outcomes and alter the disease course [18]. Within the Roma population, Kolvek et al. (2025) demonstrated a significant prevalence of autosomal recessive Alport syndrome, representing a higher risk compared to Slovakia’s majority population [19].

At the outpatient genetic clinic in the Children’s Faculty Hospital in Košice, Eastern Slovakia, we identified the most frequent rare neurodevelopmental genetic disorders that primarily affect the Roma community—largely due to the founder effect. Our objective was to conduct a comprehensive analysis of all cases of these rare diseases that were diagnosed at the Medical Genetics and Metabolic Outpatient Clinic during the five-year period from 2019 to 2025.

## 2. Materials and Methods

### 2.1. Study Design and Ethical Considerations

This retrospective study was conducted between January 2019 and January 2025 at the outpatient clinic at the Eastern Slovakia Clinical Genetics and Metabolics department at the Children’s University Hospital, Košice. This outpatient clinic cares for all child patients with genetic disorders in the Kosice region from the newborn period to young adulthood. Patients were diagnosed via molecular–genetic methods in private laboratories. For the detection of specific variants, we performed targeted gene sequencing using Sanger sequencing (for validation and known hotspot regions) in most cases, and in a few cases we employed Next-Generation Sequencing (NGS)–based whole-exome sequencing (WES). This study was approved by the Ethics Committee of the Children’s University Hospital in Košice (approval number 07/2025). All patients—or their parents, when applicable—had previously provided consent to participate. The requirement for obtaining new written informed consent was waived in accordance with ICH-GCP 135/95 guidelines due to the retrospective design of this study and the use of de-identified clinical data. The authors confirm that no patient in this cohort had opted out of, or explicitly refused, the use of their medical records for research purposes.

### 2.2. Participants

This study included 61 patients, ranging from 1.5 months to 25 years of age, who were diagnosed with hypomyelinating leukodystrophy 14, pontocerebellar hypoplasia type 1B, neuronal ceroid lipofuscinosis 7, or TMEM70 deficiency at the Outpatient Clinic of the Clinical Genetics and Metabolics Department in Eastern Slovakia. We extracted clinical, demographic, genetic, laboratory, and neuroimaging data from hospital records, including age at diagnosis, sex, neurological findings, genetic test results, survival status, and any available family history. Although many other rare disorders are diagnosed at our department, this analysis focused on four recurrent neurodevelopmental conditions frequently observed in the Roma community. Complete pedigree information was unavailable for most families, preventing accurate classification of simplex versus multiplex cases and precluding pedigree-based assessment of consanguinity. No haplotype or identity-by-descent analyses were performed; however, all affected individuals were homozygous for their respective pathogenic variants, each of which is a recognized founder mutation in the Roma population. Data were collected between 2019 and 2025.

### 2.3. Statistical Analyses

First, we summarized demographic variables, genetic diagnoses, and clinical characteristics using descriptive statistics (frequencies, percentages, means, and ranges). Second, we classified each disorder according to its molecular etiology and used SPSS version 23.0 to perform all statistical analyses. Because this study involved a relatively small sample size and categorical clinical variables, we applied non-parametric methods, including the Chi-square test or Fisher’s exact test (when expected cell counts were <5), to compare proportions across diagnostic groups.

## 3. Results

**Figure 1:**
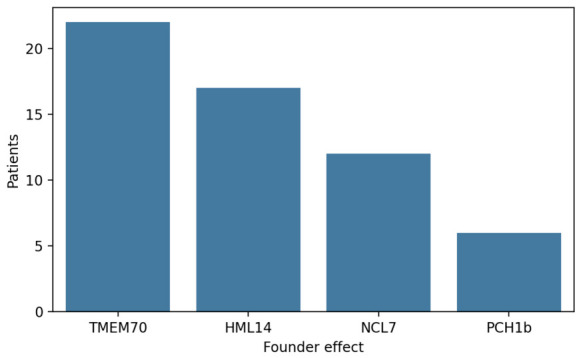
Distribution of patients affected by four founder-related genetic disorders in the Roma population.

This study included a total of 61 patients, the majority of which were diagnosed with TMEM 70 deficiency (*n* = 25). The cohort also included patients with hypomyelinating leukodystrophy (*n* = 17), neuronal ceroid lipofuscinosis (*n* = 13), and pontocerebellar hypoplasia type 1B (*n* = 6) (Figure 1).

### 3.1. Hypomyelinating Leukodystrophy 14

Our patient sample represents the largest known group of individuals with this specific mutation in the *UFM1* gene (c.-273_-271delTCA), comprising a total of 17 patients. Among these, 64% are male (*n* = 11) and 36% are female (*n* = 6). Diagnoses were established at the molecular–genetic level, with an average duration of 5.7 months from the onset of clinical symptoms to diagnosis. The *UFM1* c.-273_-271delTCA variant corresponds to NM_001286704.1 and is located at chr13:38349765–38349767 (GRCh38). Because the negative position indicates a non-coding change in the 5′ promoter region, its functional consequence involves disruption of regulatory elements required for transcription. Functional studies have shown that this promoter deletion significantly reduces UFM1 expression, confirming its pathogenicity. This variant is a well-documented Roma founder mutation, previously reported in multiple affected families and classified as pathogenic in ClinVar. This mutation is associated with a severe neurodegenerative disease characterized by high mortality rates, often occurring before the first year of life. In our cohort, nearly all patients have passed away, with only one patient currently alive. The average age at death was 11.8 months, and the longest-surviving patient lived to be 30 months old. All patients presented with several clinical manifestations, including inspiratory laryngeal stridor, axial hypotonia, spastic quadriparesis, pseudobulbar syndrome, and microcephaly (Table 1).

### 3.2. Pontocerebellar Hypoplasia Type 1B

Our patient sample includes six Roma individuals, equally divided between boys and girls, all of whom share the same mutation in the *EXOSC3* gene (c.92G>C) and exhibit high levels of consanguinity. The *EXOSC3* variant identified in our cohort corresponds to NM_016042.4:c.92G>C, resulting in the missense substitution p.Gly31Ala at the protein level (NP_057126.2). This pathogenic variant is located on chromosome 9p13.2, with genomic coordinates chr9:37,784,953 (GRCh38) and chr9:37,784,950 (GRCh37). It is listed in ClinVar (Variation ID: 31691) as pathogenic/likely pathogenic with multiple independent submitters and no conflicting interpretations. The average age at which a diagnosis was established was 8.8 months. Unfortunately, almost all patients passed away by the age of three, with an average age of 16.8 months at the time of death. Each presented with microcephaly, severe global hypotonia, and spastic quadriplegia (Table 2).

### 3.3. Neuronal Ceroid Lipofuscinosis 7

Our patient sample included 13 children from eight families with reported consanguinity, all sharing the same mutation in the *MFSD8* gene (c.881C>A). The *MFSD8* variant identified in our cohort corresponds to NM_152778.3:c.881C>A. The *MFSD8* gene maps to chr4:127,917,732–127,965,963 (GRCh38) and encodes a lysosomal transporter implicated in neuronal ceroid lipofuscinosis 7. This specific variant is not listed in public databases such as ClinVar or gnomAD, and therefore no standardized genomic coordinates, protein-level consequence, or in silico pathogenicity predictions are available. Its recurrence in multiple affected children, absence from population databases, and phenotype consistency support its pathogenicity. A regression of psychomotor development was observed between the ages of 3 and 4 years, and 54% of boys (*n* = 7) and 46% of girls (*n* = 6) exhibited symptoms of intellectual disability, tremor, clumsiness, ataxia, and cerebral seizures. The genetic diagnoses were established at an average age of 4.5 years. Currently, five children are still living, while the others passed away at an average age of 8.6 years (Table 3).

### 3.4. TMEM70 Deficiency

In our small region, we identified 25 patients with a typical mutation in the *TMEM70* gene (c.317-2A>G), associated with the Roma ethnicity and characteristic symptoms. The *TMEM70* variant identified in our patients corresponds to NM_017866.6:c.317-2A>G, a pathogenic splice-acceptor mutation located at chr8:73981153A>G (GRCh38). This variant disrupts ATP synthase assembly and is a recognized cause of mitochondrial Complex V deficiency. ClinVar classifies this specific variant (c.317-2A>G) as pathogenic, supported by multiple independent submitters and no conflicting interpretations. Within our sample, 72% were girls (*n* = 18) and 28% (*n* = 7) were boys, with an average age of 9.95 years. Notably, the oldest living patient is an impressive 25 years old. Unfortunately, four patients passed away due to severe metabolic crises. All patients suffer from intellectual disability, global hypotonia, hypertrophic cardiomyopathy, epilepsy, and failure to thrive (Table 4).

## 4. Discussion

**Figure 2:**
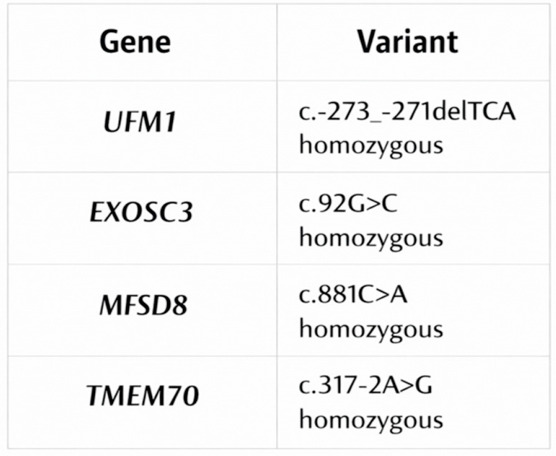
Pathogenic homozygous variants identified in the study cohort.

Due to the unique demographic history of the Romani community, several genetic diseases occur more frequently in this population than in non-Roma groups. As part of efforts to better understand these patterns, genomic studies have identified numerous novel mutations associated with specific monogenic disorders in Roma individuals. This is largely explained by genetic drift, a random process that alters allele and genotype frequencies over time, particularly in small, isolated, or endogamous populations [20]. The Roma population illustrates how inherited disorders can become more common in groups that originate from a small number of ancestors. In our region, the rare neurodevelopmental conditions most frequently observed in the Roma community include hypomyelinating leukodystrophy 14, pontocerebellar hypoplasia type 1B, neuronal ceroid lipofuscinosis 7, and TMEM70 deficiency with their variants (Figure 2). Their increased occurrence is linked to the historical demographic structure of the Roma population, which has led to a higher concentration of specific pathogenic variants.

Hypomyelinating leukodystrophy type 14 is a rare and relatively new neurodevelopmental disorder that occurs mainly in the Romani ethnic group. This disorder is caused by a pathogenic variant of the *UFM1* gene located on chromosome 13q13. Ubiquitination involves the attachment of ubiquitin molecules to proteins to control various cellular functions. The ubiquitin-like modifier (UFM-1) can connect to proteins as a monomer or polymer linked to lysine. UFMylation is a specific modification that supports the production of metazoan proteins in the endoplasmic reticulum and is vital for the nervous system’s development and function. Defective UFMylation is linked to severe neurodevelopmental issues seen in HLD14. The disease is transmitted in an autosomal recessive manner and begins in early childhood [21]. In 2017, Hamilton et al. identified 16 pediatric patients with a severe clinical phenotype of epileptic encephalopathy and psychomotor retardation with typical brain MRI findings of hypomyelination with basal ganglia and cerebellar atrophy. The majority of these patients were of Romani ancestry [22]. In 2018, Nahorski et al. reported four children with a homozygous missense mutation in the *UFM1* gene who died before the age of two [21]. Our patient sample is the largest known cohort with the *UFM1* gene mutation (c.-273_-271delTCA), comprising 17 individuals, predominantly males. We noted a higher mortality rate occurring before the age of one year. This neurodegenerative disease is associated with significant morbidity, as all patients exhibited symptoms such as inspiratory laryngeal stridor, axial hypotonia, spastic quadriparesis, pseudobulbar syndrome, and microcephaly. The incidence of this condition is high in our region, and the average duration from symptom onset to diagnosis is 5.7 months. The screening of 1000 controls from various European Roma panels revealed a mutation carrier rate ranging from 3% to as high as 25% [22].

Pontocerebellar hypoplasia type 1b (PCH1B) is caused by a mutation in the *EXOSC3* gene, which is located on chromosome 9p13 and can be either homozygous or compound heterozygous. Mutations in genes associated with PCH can impact the production of enzymes that are vital for the development of nerve cells (neurons) and for the proper processing of RNA, which plays a critical role in the normal functioning of all cells. However, the precise mechanisms through which PCH affects the development of the cerebellum and pons remain an area for further exploration and understanding. Typical clinical features include diffuse muscle weakness, progressive microcephaly, global developmental delay, and the involvement of the brainstem [23]. In 2013, Schwabová et al. [24] identified a homozygous mutation, c.92G>C (p.G31A), in the *EXOSC3* gene in 32 Czech children of Romani descent with PCH1B. A heterozygous mutation was identified in 4 out of 90 unrelated Romani controls (4.4%), suggesting a founder effect. The affected patients had a severe form of the disorder, often resulting in death within the first year of life [24]. The patient cohort consisted of six Roma individuals, evenly divided by gender, who all possessed the same mutation in the *EXOSC3* gene. The average age at which a diagnosis was established was 8.8 months. Our findings indicate a higher mortality rate; the majority of patients had died by the age of three. Additionally, we observed a significant mortality rate in cases presenting with microcephaly, severe global hypotonia, and spastic quadriplegia.

Neuronal ceroid lipofuscinosis type 7 (CLN7) is an autosomal recessive disease that results from homozygous or biallelic heterozygous variants in the *MFSD8* gene. This gene is responsible for encoding a versatile lysosomal transmembrane protein that is composed of 518 amino acids and features 12 membrane-spanning domains. Mutations that affect the function of MFSD8 are associated with a lysosomal storage disorder known as CLN7 disease and are part of the neuronal ceroid lipofuscinosis group (NCL). This condition is characterized by a progressive decline in neuronal health, alongside the occurrence of seizures and cognitive challenges [25]. In a study involving 14 Roma patients from 12 families with NCL, Kousi et al. (2009) identified a homozygous transversion c.881C>A (p.Thr294Lys) located in exon 10 of the *MFSD8* gene [26]. Notably, seven of these patients originated from the former Czechoslovakia. The patients exhibited a phenotype characterized by a late onset in childhood, developmental regression, seizures, visual impairments, and ataxia. A haplotype analysis in Roma patients indicated a founder effect [26]. The patient sample comprised 13 children from eight families, all of whom presented the same mutation in the *MFSD8* gene. Notably, regression in psychomotor development was observed from the ages of 3 to 4 years. Symptoms such as intellectual disability, tremors, clumsiness, ataxia, and cerebral seizures were predominantly identified in males compared to females. The average age for establishing a genetic diagnosis was 4.5 years. Additionally, a concerning mortality rate was observed, as eight children died before reaching the age of 9 years.

Inherited ATP synthase disorders are severe mitochondrial diseases that primarily affect children [27,28]. TMEM70 deficiency is increasingly recognized as one of the most common mitochondrial disorders in pediatric populations. Mitochondrial ATP synthase plays an essential role in the process of energy production within the mitochondria, as it facilitates the synthesis of ATP during oxidative phosphorylation. Its importance in cellular energy metabolism cannot be overstated. A mutation in the *TMEM 70* gene adversely affects the assembly of mitochondrial ATP synthase (Complex V), leading to a reduction in ATP production. Since its identification in 2008, over 50 cases have been documented. A 2006 study reported 14 patients with symptoms such as hypertrophic cardiomyopathy, hypotonia, and psychomotor delays [29]. In 2010, 25 children with the c.317-2A > G mutation were identified as exhibiting severe disease onset. More cases of TMEM70 deficiency have since been identified across diverse ethnic backgrounds, presenting a variety of phenotypes and disease progressions [30]. In 2014, Magner et al. characterized the natural history, outcomes, and clinical experiences of therapy in patients with mutations in the *TMEM70* gene. The study involved 11 centers across eight European countries, as well as Turkey and Israel. A total of 48 patients (26 boys and 22 girls) from 38 families were enrolled, making this the largest patient cohort worldwide with this condition [31]. In our designated region, we identified 25 patients with a specific mutation in the *TMEM70* gene, which is linked to the Roma ethnicity and presents with characteristic symptoms. Within this cohort, the gender distribution reveals a predominance of females over males, with an average age of 9.95 years. Remarkably, the oldest living patient was 25 years of age. Regrettably, four patients died to complications arising from severe metabolic crises. Our observations indicate a heightened prevalence of morbidity, which includes intellectual disability, global hypotonia, hypertrophic cardiomyopathy, epilepsy, and failure to thrive.

Another key factor influencing the gene pool of the Roma population is the founder effect. Migration has led to isolated subpopulations, in which a secondary founder effect has shaped the modern Roma community, creating genetically distinct sub-isolates. Additionally, the establishment of smaller groups and communities has contributed to a tertiary founder effect. These effects explain the differing frequencies of DNA variants within different Roma sub-isolates and contribute to a shared presence of hereditary diseases and unique mutations in their gene pool [32,33].

### Limitations of the Study

This study has several limitations. First, its retrospective design inherently limits the completeness and uniformity of clinical and family-history data. Information on consanguinity, pedigree structure, and extended familial relationships was unavailable for most families, preventing a reliable assessment of inheritance patterns or quantification of consanguinity. Second, although all patients carried identical pathogenic variants consistent with known founder mutations in the Roma population, no haplotype or identity-by-descent analyses were performed to genetically confirm a shared ancestral origin. Third, detailed demographic data, environmental factors, and access to healthcare services were not systematically captured, which may influence both diagnostic timing and phenotypic severity. Finally, because this cohort was recruited from a single clinical genetics center in Eastern Slovakia, the findings may not fully represent all Roma subpopulations across the region. Despite these limitations, the study provides valuable insight into the burden and clinical presentation of recurrent monogenic neurodevelopmental disorders in this underserved population.

## 5. Conclusions

Most rare diseases have a genetic origin, meaning they are present throughout a person’s life, even if symptoms do not manifest immediately. These diseases are characterized by high morbidity and mortality rates. At present, there is no targeted therapy available for any of these neurodevelopmental diseases. Elevated levels of consanguinity increase the likelihood that both parents carry the same recessive gene. This situation results in a higher prevalence of autosomal recessive diseases. The implementation of prenatal and newborn screening programs for the most prevalent diseases within the community is essential. Additionally, voluntary carrier testing for couples prior to family planning should be emphasized as a vital component of proactive health management.

## Figures and Tables

**Table 1:** Characteristics of patients with hypomyelinating leukodystrophy 14 (MIM number 617899).

Patients	Gender	Genetic	Inheritance	Age of Diagnosis (m = Month)	Exitus (m = Month)	Type	Signs
Patient 1	M	*UFM1* (c.-273_-271delTCA) homozygous	AR	7	Alive	HL14	Inspiratory laryngeal stridor, axial hypotonia, spastic kvadruparesis, pseudobulbar syndrome, microcephaly
Patient 2	F	*UFM1* (c.-273_-271delTCA) homozygous	AR	4	15	HL14	Inspiratory laryngeal stridor, axial hypotonia, spastic kvadruparesis, horizontal nystagmus, pseudobulbar syndrome, microcephaly
Patient 3	F	*UFM1* (c.-273_-271delTCA) homozygous	AR	5	9	HL14	Inspiratory laryngeal stridor, axial hypotonia, spastic kvadruparesis, horizontal nystagmus, pseudobulbar syndrome, microcephaly
Patient 4	M	*UFM1* (c.-273_-271delTCA) homozygous	AR	2	3	HL14	Inspiratory laryngeal stridor, axial hypotonia, spastic kvadruparesis, pseudobulbar syndrome, microcephaly
Patient 5	M	*UFM1* (c.-273_-271delTCA) homozygous	AR	3	5	HL14	Inspiratory laryngeal stridor, axial hypotonia, spastic kvadruparesis, pseudobulbar syndrome, microcephaly
Patient 6	F	*UFM1* (c.-273_-271delTCA) homozygous	AR	8	9	HL14	Inspiratory laryngeal stridor, axial hypotonia, spastic kvadruparesis, pseudobulbar syndrome
Patient 7	F	*UFM1* (c.-273_-271delTCA) homozygous	AR	5	8	HL14	Inspiratory laryngeal stridor, axial hypotonia, spastic kvadruparesis, pseudobulbar syndrome, horizontal nystagmus, microcephaly
Patient 8	M	*UFM1* (c.-273_-271delTCA) homozygous	AR	7	22	HL14	Inspiratory laryngeal stridor, axial hypotonia, spastic kvadruparesis, pseudobulbar syndrome
Patient 9	M	*UFM1* (c.-273_-271delTCA) homozygous	AR	5	16	HL14	Inspiratory laryngeal stridor, axial hypotonia, spastic kvadruparesis, pseudobulbar syndrome
Patient 10	M	*UFM1* (c.-273_-271delTCA) homozygous	AR	6	8	HL14	Inspiratory laryngeal stridor, axial hypotonia, spastic kvadruparesis, pseudobulbar syndrome
Patient 11	F	*UFM1* (c.-273_-271delTCA) homozygous	AR	6	12	HL14	Inspiratory laryngeal stridor, axial hypotonia, spastic kvadruparesis, pseudobulbar syndrome, microcephaly
Patient 12	M	*UFM1* (c.-273_-271delTCA) homozygous	AR	5	12	HL14	Inspiratory laryngeal stridor, axial hypotonia, spastic kvadruparesis, pseudobulbar syndrome, microcephaly
Patient 13	M	*UFM1* (c.-273_-271delTCA) homozygous	AR	5	6	HL14	Inspiratory laryngeal stridor, axial hypotonia, spastic kvadruparesis, pseudobulbar syndrome, microcephaly
Patient 14	M	*UFM1* (c.-273_-271delTCA) homozygous	AR	8	10	HL14	Inspiratory laryngeal stridor (tracheostoma), axial hypotonia, spastic kvadruparesis, pseudobulbar syndrome, microcephaly
Patient 15	M	*UFM1* (c.-273_-271delTCA) homozygous	AR	3	11	HL14	Inspiratory laryngeal stridor, axial hypotonia, spastic kvadruparesis, pseudobulbar syndrome, microcephaly
Patient 16	M	*UFM1* (c.-273_-271delTCA) homozygous	AR	8	14	HL14	Inspiratory laryngeal stridor, axial hypotonia, spastic kvadruparesis, pseudobulbar syndrome, microcephaly
Patient 17	F	*UFM1* (c.-273_-271delTCA) homozygous	AR	5	30	HL14	Inspiratory laryngeal stridor, axial hypotonia, spastic kvadruparesis, pseudobulbar syndrome, microcephaly

**Table 2:** Characteristics of patients with pontocerebellar hypoplasia type 1B (MIM number 614678).

Patients	Genre	Genetic	Inheritance	Age of Diagnosis (m = Month)	Exitus (m = Month)	Type	Signs
Patient 1	F	*EXOSC3* (c.92G>C) homozygous	AR	30	36	PCH1b	Hypoxic–ischemic encephalopathy, microcephaly, spastic quadriplegia, global hypotonia, cortico-subcortical atrophy
Patient 2	M	*EXOSC3* (c.92G>C) homozygous	AR	9	12	PCH1b	Inspiratory laryngeal stridor, microcephaly, global hypotonia, milestone progress not reached
Patient 3	M	*EXOSC3* (c.92G>C) homozygous	AR	3	7	PCH1b	Microcephaly, global hypotonia, spastic quadriplegia, ASD II, multiorgan failure, pseudo-oedema of limbs
Patient 4	F	*EXOSC3* (c.92G>C) homozygous	AR	8	24	PCH1b	Microcephaly, global hypotonia, milestone progress not reached, pseudo-oedema of limbs
Patient 5	F	*EXOSC3* (c.92G>C) homozygous	AR	1.5	5	PCH1b	Inspiratory laryngeal stridor, axial hypotonia, spastic quadriplegia, enophtalmus, dystonia, seizures, microcephaly
Patient 6	M	*EXOSC3* (c.92G>C) homozygous	AR	1.5	alive	PCH1b	Inspiratory laryngeal stridor, axial hypotonia, spastic quadriplegia, dystonia, seizures, microcephaly

**Table 3:** Characteristics of patients with neuronal ceroid lipofuscinosis 7 (MIM number 610951).

Patients	Gender	Genetic	Inheritance	Age of Diagnosis (Y = Year)	Exitus/Actual Age (Y = Year)	Type	Signs
Patient 1	F	*MFSD8* (c. 881C>A) homozygous	AR	5	alive	CLN7	Loss of developed skills in 3rd year of life, intellectual disability, tremor, clumsiness, ataxia, cerebral seizures (myoclonic)
Patient 2	F	*MFSD8* (c. 881C>A) homozygous	AR	4	alive	CLN7	Loss of developed skills in 3rd year of life, microcephaly, intellectual disability, ataxia, clumsiness
Patient 3	M	*MFSD8* (c. 881C>A) homozygous	AR	5	alive	CLN7	Loss of developed skills in 3rd year of life, microcephaly, intellectual disability, ataxia, clumsiness
Patient 4	M	*MFSD8* (c. 881C>A) homozygous	AR	4	alive	CLN7	Loss of developed skills in 3rd year of life, intellectual disability, ataxia, clumsiness, cerebral seizures (myoclonic)
Patient 5	F	*MFSD8* (c. 881C>A) Homozygous	AR	4	8	CLN7	Loss of developed skills in 3rd year of life, intellectual disability, ataxia, clumsiness, cerebral seizures (myoclonic)
Patient 6	M	*MFSD8* (c. 881C>A) Homozygous	AR	3.5	alive	CLN7	Loss of developed skills in 3rd year of life, intellectual disability, ataxia, clumsiness, cerebral seizures (myoclonic)
Patient 7	M	*MFSD8* (c. 881C>A) Homozygous	AR	7	9	CLN7	Loss of developed skills in 3rd year of life, intellectual disability, ataxia, clumsiness, pseudobulbar syndrome, blindness, cerebral seizures
Patient 8	F	*MFSD8* (c. 881C>A) Homozygous	AR	4	8	CLN7	Loss of developed skills in 3rd year of life, intellectual disability, ataxia, clumsiness, pseudobulbar syndrome, vision impairment, cerebral seizures
Patient 9	F	*MFSD8* (c. 881C>A) Homozygous	AR	5	7	CLN7	Loss of developed skills in 3.5rd year of life, intellectual disability, ataxia, clumsiness, pseudobulbar syndrome, blindness, cerebral seizures
Patient 10	F	*MFSD8* (c. 881C>A) Homozygous	AR	4	11	CLN7	Loss of developed skills in 3rd year of life, intellectual disability, ataxia, clumsiness, pseudobulbar syndrome, vision impairment
Patient 11	M	*MFSD8* (c. 881C>A) Homozygous	AR	4	8	CLN7	Loss of developed skills in 4th year of life, intellectual disability, ataxia, clumsiness, pseudobulbar syndrome, blindness, cerebral seizures
Patient 12	M	*MFSD8* (c. 881C>A) Homozygous	AR	5	8	CLN7	Loss of developed skills in 4th year of life, intellectual disability, ataxia, clumsiness, pseudobulbar syndrome, blindness, cerebral seizures
Patient 13	M	*MFSD8* (c. 881C>A) homozygous	AR	4	10	CLN7	Loss of developed skills in 3rd year of life, intellectual disability, ataxia, clumsiness, pseudobulbar syndrome, blindness, cerebral seizures

**Table 4:** Characteristics of patients with TMEM70 deficiency (MIM number 612418).

Patients	Gender	Genetic	Inheritance	Exitus/Actual Age (Y = Year)	Type	Signs
Patient 1	F	*TMEM70* (c.317-2A>G) homozygous	AR	11	TMEM70 deficiency	Intellectual disability, global hypotonia, hypertrophic cardiomyopathy, epilepsy
Patient 2	F	*TMEM70* (c.317-2A>G) homozygous	AR	25	TMEM70 deficiency	Intellectual disability, global hypotonia, hypertrophic cardiomyopathy, epilepsy, strabism, short statue
Patient 3	F	*TMEM70* (c.317-2A>G) homozygous	AR	11	TMEM70 deficiency	Intellectual disability, microcephaly, global hypotonia, hypertrophic cardiomyopathy, epilepsy, malnutrition
Patient 4	F	*TMEM70* (c.317-2A>G) homozygous	AR	7	TMEM70 deficiency	Intellectual disability, global hypotonia, hypertrophic cardiomyopathy, short statue
Patient 5	M	*TMEM70* (c.317-2A>G) homozygous	AR	4	TMEM70 deficiency	Intellectual disability, global hypotonia, hypertrophic cardiomyopathy, epilepsy, short statue, strabism
Patient 6	F	*TMEM70* (c.317-2A>G) homozygous	AR	13	TMEM70 deficiency	Intellectual disability, global hypotonia, hypertrophic cardiomyopathy, short statue
Patient 7	M	*TMEM70* (c.317-2A>G) homozygous	AR	8	TMEM70 deficiency	Intellectual disability, global hypotonia, hypertrophic cardiomyopathy, short statue, malnutrition
Patient 8	F	*TMEM70* (c.317-2A>G) homozygous	AR	9	TMEM70 deficiency	Intellectual disability, global hypotonia, hypertrophic cardiomyopathy, short statue. malnutrition
Patient 9	F	*TMEM70* (c.317-2A>G) homozygous	AR	Exitus	TMEM70 deficiency	Neonatal encephalocardiomyopathy, metabolic crisis
Patient 10	M	*TMEM70* (c.317-2A>G) homozygous	AR	Exitus	TMEM70 deficiency	Neonatal encephalocardiomyopathy, exitus of metabolic crisis
Patient 11	M	*TMEM70* (c.317-2A>G) homozygous	AR	5	TMEM70 deficiency	Intellectual disability, global hypotonia, hypertrophic cardiomyopathy, short statue
Patient 12	F	*TMEM70* (c.317-2A>G) Homozygous	AR	5	TMEM70 deficiency	Intellectual disability, global hypotonia, hypertrophic cardiomyopathy, short statue, polydactylia, strabism, short statue
Patient 13	F	*TMEM70* (c.317-2A>G) Homozygous	AR	Exitus	TMEM70 deficiency	Intellectual disability, global hypotonia, hypertrophic cardiomyopathy, short statue, malnutrition
Patient 14	F	*TMEM70* (c.317-2A>G) Homozygous	AR	6	TMEM70 deficiency	Intellectual disability, global hypotonia, hypertrophic cardiomyopathy, short statue, malnutrition
Patient 15	M	*TMEM70* (c.317-2A>G) Homozygous	AR	20	TMEM70 deficiency	Intellectual disability, global hypotonia, hypertrophic cardiomyopathy, short statue, malnutrition
Patient 16	M	*TMEM70* (c.317-2A>G) Homozygous	AR	8	TMEM70 deficiency	Intellectual disability, global hypotonia, hypertrophic cardiomyopathy, short statue, malnutrition
Patient 17	F	*TMEM70* (c.317-2A>G) Homozygous	AR	8	TMEM70 deficiency	Intellectual disability, global hypotonia, hypertrophic cardiomyopathy, short statue, malnutrition
Patient 18	F	*TMEM70* (c.317-2A>G) Homozygous	AR	21	TMEM70 deficiency	Intellectual disability, global hypotonia, hypertrophic cardiomyopathy, short statue, malnutrition
Patient 19	F	*TMEM70* (c.317-2A>G) Homozygous	AR	Exitus	TMEM70 deficiency	Neonatal encephalocardiomyopathy
Patient 20	F	*TMEM70* (c.317-2A>G) Homozygous	AR	20	TMEM70 deficiency	Intellectual disability, global hypotonia, hypertrophic cardiomyopathy, short statue, malnutrition
Patient 21	M	*TMEM70* (c.317-2A>G) Homozygous	AR	Exitus	TMEM70 deficiency	Neonatal encephalocardiomyopathy
Patient 22	F	*TMEM70* (c.317-2A>G) Homozygous	AR	10	TMEM70 deficiency	Intellectual disability, global hypotonia, hypertrophic cardiomyopathy, short statue, malnutrition
Patient 23	F	*TMEM70* (c.317-2A>G) Homozygous	AR	1	TMEM70 deficiency	Intellectual disability, global hypotonia, hypertrophic cardiomyopathy, short statue, malnutrition
Patient 24	F	*TMEM70* (c.317-2A>G) Homozygous	AR	5	TMEM70 deficiency	Intellectual disability, global hypotonia, hypertrophic cardiomyopathy, short statue, malnutrition
Patient 25	F	*TMEM70* (c.317-2A>G) homozygous	AR	2	TMEM70 deficiency	Intellectual disability, global hypotonia, hypertrophic cardiomyopathy, short statue, malnutrition

## Data Availability

No datasets were generated or analyzed during the current study.
